# Determination of wheat spike and spikelet architecture and grain traits using X-ray Computed Tomography imaging

**DOI:** 10.1186/s13007-021-00726-5

**Published:** 2021-03-09

**Authors:** Hu Zhou, Andrew B. Riche, Malcolm J. Hawkesford, William R. Whalley, Brian S. Atkinson, Craig J. Sturrock, Sacha J. Mooney

**Affiliations:** 1 School of Biosciences, University of Nottingham, Sutton Bonington Campus, Loughborough, LE12 5RD Leicestershire UK; 2 Rothamsted Research, Harpenden, AL5 2JQ UK

**Keywords:** Wheat ear, Wheat grain, X-ray microtomography

## Abstract

**Background:**

Wheat spike architecture is a key determinant of multiple grain yield components and detailed examination of spike morphometric traits is beneficial to explain wheat grain yield and the effects of differing agronomy and genetics. However, quantification of spike morphometric traits has been very limited because it relies on time-consuming manual measurements.

**Results:**

In this study, using X-ray Computed Tomography imaging, we proposed a method to efficiently detect the 3D architecture of wheat spikes and component spikelets by clustering grains based on their Euclidean distance and relative positions. Morphometric characteristics of wheat spikelets and grains, e.g., number, size and spatial distribution along the spike can be determined. Two commercial wheat cultivars, one old, Maris Widgeon, and one modern, Siskin, were studied as examples. The average grain volume of Maris Widgeon and Siskin did not differ, but Siskin had more grains per spike and therefore greater total grain volume per spike. The spike length and spikelet number were not statistically different between the two cultivars. However, Siskin had a higher spikelet density (number of spikelets per unit spike length), with more grains and greater grain volume per spikelet than Maris Widgeon. Spatial distribution analysis revealed the number of grains, the average grain volume and the total grain volume of individual spikelets varied along the spike. Siskin had more grains and greater grain volumes per spikelet from spikelet 6, but not spikelet 1–5, compared with Maris Widgeon. The distribution of average grain volume along the spike was similar for the two wheat cultivars.

**Conclusion:**

The proposed method can efficiently extract spike, spikelet and grain morphometric traits of different wheat cultivars, which can contribute to a more detailed understanding of the sink of wheat grain yield.

**Supplementary Information:**

The online version contains supplementary material available at 10.1186/s13007-021-00726-5.

## Highlight

A CT imaging-based method was developed to efficiently quantify wheat spike traits.

## Background

Currently about 20% of total dietary calories and protein for the world’s human population come from wheat [2]. By 2050, food demand of the global population (predicted to be > 9 billion) will increase by around 70% [15], UN, 2017). Increasing wheat yield is therefore crucial to ensure global food security [14, 15]. Current evidence indicates that wheat yield under favourable conditions is mainly limited by sink strength [1, 20]. Analysis of breeding history also revealed wheat grain yield improvement in the last century was highly associated with increase in grain number per unit area, which is largely determined by the grain number per spike [2]. Wheat grain number per spike is determined by the combination of number of spikelets per spike and the number of grains per spikelet. In contrast to the spikelet of other cereal crops such as rice and barley, each wheat spikelet has more than one grain. This makes the wheat spikelet one of the most essential grain yield components [18]. Therefore, in-depth examinations of the morphometric traits of spikelets are fundamental to the study of their genetic loci and the breeding of high yielding wheats [19].

To date, morphometric traits of wheat spikes and spikelets have been measured manually [10], which is laborious, destructive and often results in the loss of the spatial information of the spikelet. From the literature, we recorded only one study [11] that developed a deep learning-based approach to automatically count spikelets from 2D optical images. However, because of the limitation of the 2D optical images, this method cannot provide information (e.g., size) on the grains within the spikelet. There is a critical need to develop methods to quantify morphometric traits of the wheat spike, spikelet and the grains within the spikelets.

Recently, X-ray CT imaging was used to non-destructively study wheat spike and grain traits [3–5, 13, 16]. The morphometric traits of spike and grain, such as grain number, size, length, width and depth, can be extracted from their 3D images using the developed pipeline [3, 13]. Using the X-ray CT imaging technique, Hughes et al. [4] found the grains of domesticated wheat and barley had different morphometric parameters (length, width and depth) from their wild relatives. Schmidt et al. [13] found wheat grain size and number were reduced under heat and drought stress. Despite the successful use of X-ray CT imaging in the phenotyping of wheat spikes and grains, no success has been achieved to extract specifically the spikelet information, which requires consideration of the spatial arrangement of the grains.

In this study, based on the X-ray CT imaging technique, we aim to develop a new method to quantify the morphometric traits of the wheat spike, as well as wheat spikelets and individual grains. The accuracy and efficiency of the method will be discussed. To our knowledge, this is the first work on the analysis of the wheat spike and spikelet architecture in 3D using X-ray CT imaging.

## Results

### Validation of the CT imaging-based method to quantify wheat spike and spikelet traits

**Fig. 1:**
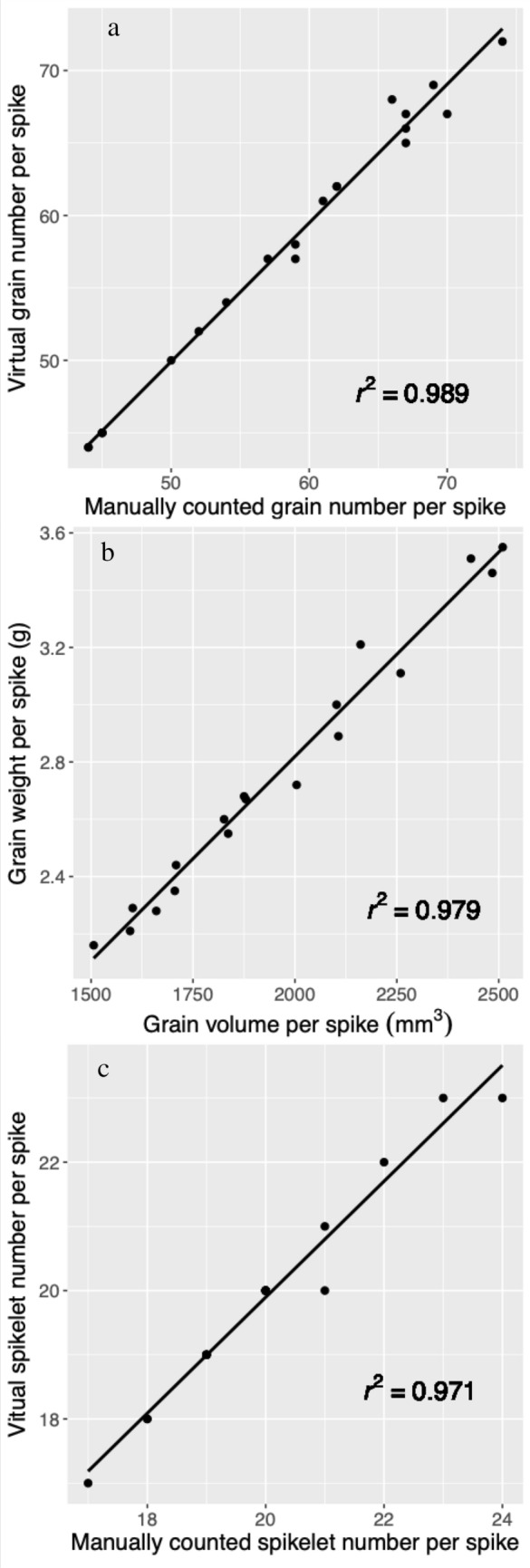
Validation of the CT imaging-based method using a manual counting-based method. **a** grain number, **b** relationship between volume and mass of grains per spike, **c** spikelet number

**Fig. 2:**
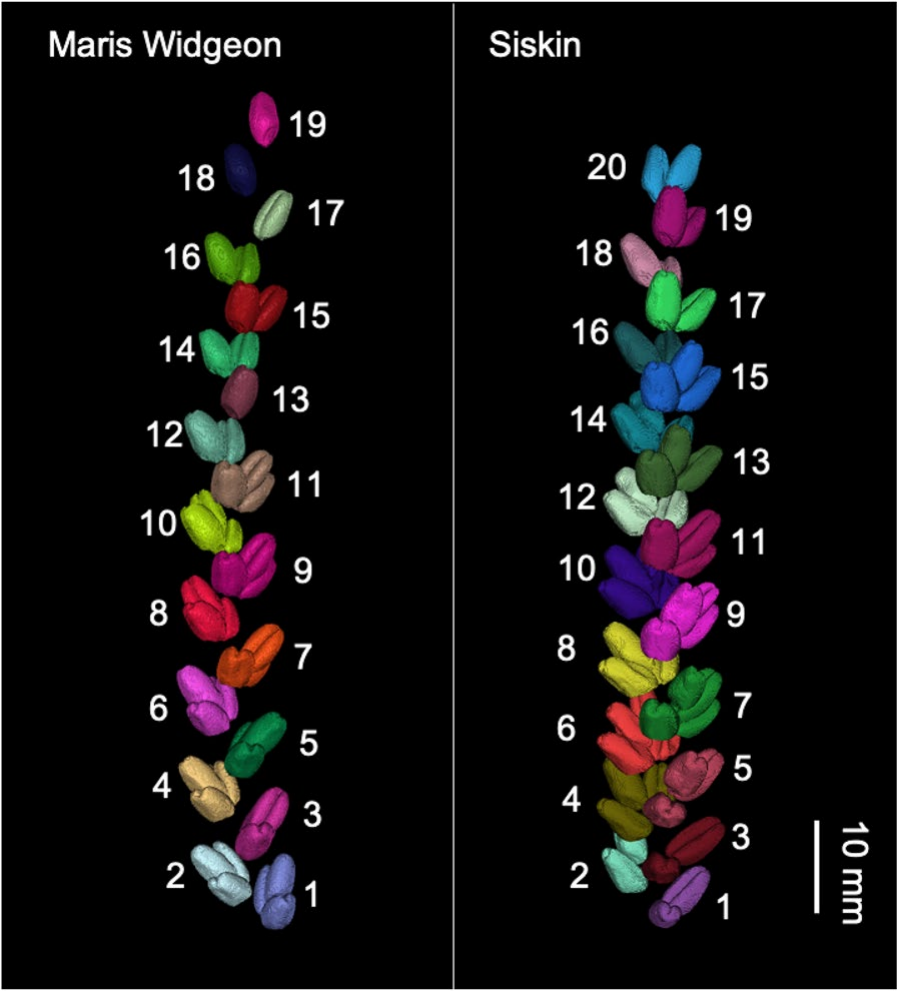
Three-dimensional visualization of wheat spikelet distribution along the spike of Maris Widgeon (left) and Siskin (right). Grains of the same spikelet shown in the same colour. Numbers indicate the spikelet sequence starting from the bottom to top along the spike

Grains number per spike determined using the CT imaging-based method was significantly correlated with the manual counting method (*r*^2^ = 0.989) (Fig. 1a). The discrepancy between the ‘virtual’ grain number and the manually counted grain number was probably caused by small grains that were filtered-out during image analysis or erroneously omitted during manually counting, which has been discussed by Hughes et al. [3] and Schmidt et al. [13]. The total grain volume of each spike was positively correlated with their mass (*r*^2^ = 0.979, Fig. 1b). The developed CT method was able to identify wheat spikelets as visualized in Fig. 2, with grains in the same spikelet shown in the same colour. The number of spikelets per spike was also well correlated with the manual method (*r*^2^ = 0.971, Fig. 1c).

### Spike morphometric traits of two wheat varieties

**Fig. 3:**
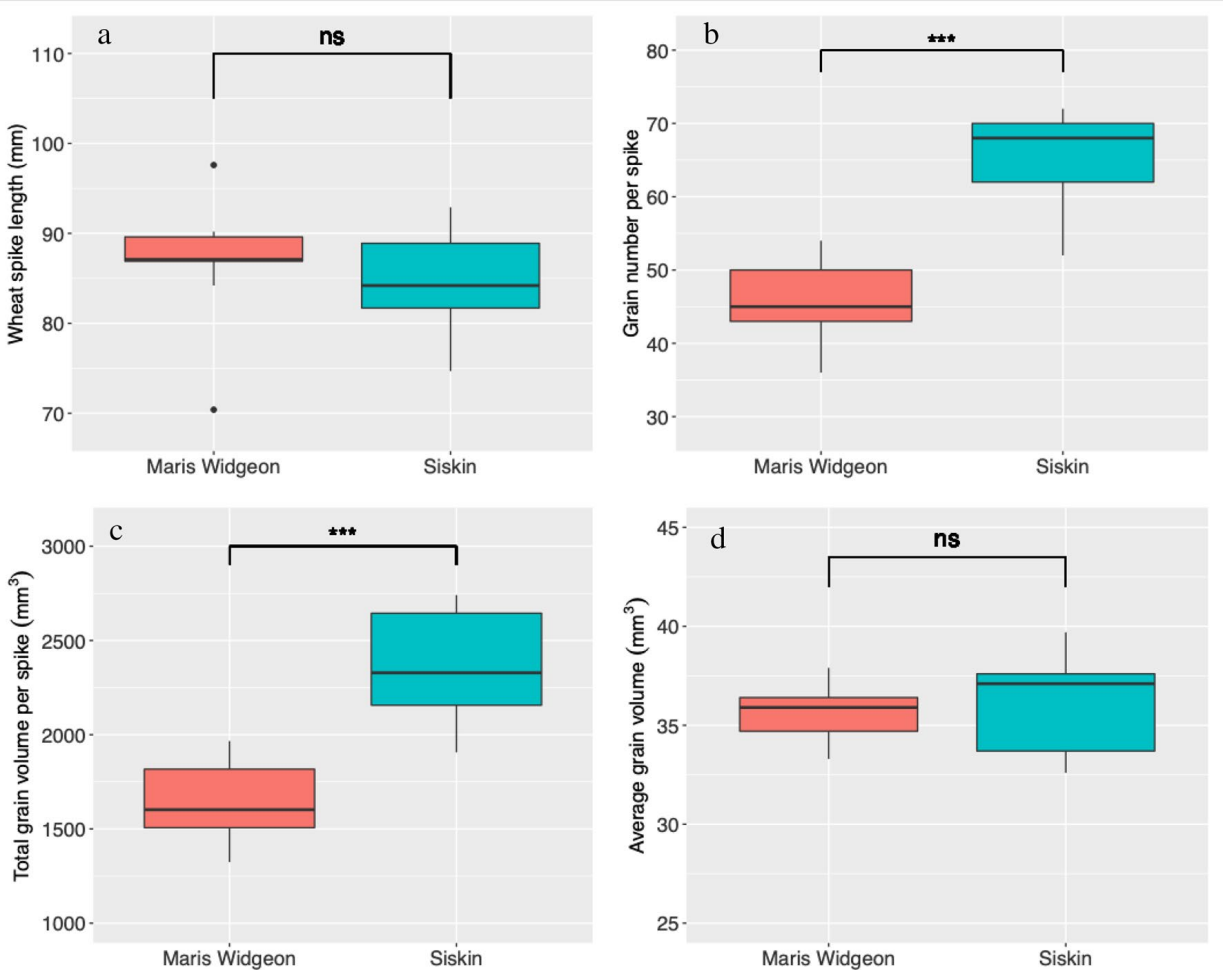
Spike morphometric traits of the two wheat varieties, Maris Widgeon and Siskin. **a** wheat spike length, **b** grain number per spike, **c** total grain volume per spike, **d** average grain volume. *ns* not significant, *P* > 0.05; ***, *P* < 0.001

Representative 3D images of wheat spikes are presented in Fig. 2. The spike length of Maris Widgeon and Siskin was 86.9 ± 7.2 and 84.8 ± 5.6 mm, respectively, without statistical difference (Fig. 3a). The Maris Widgeon had 46.3 ± 5.8 grains per spike, significantly lower than the Siskin (64.8 ± 7.0) (*P* < 0.001, Fig. 3b). The total grain volume per spike of Siskin (2331.0 ± 313.7 mm^3^) was significantly greater than that of Maris Widgeon (1648.2 ± 222.2 mm^3^) (*P* < 0.001, Fig. 3c). The average grain volume, however, did not differ between Siskin (36.0 ± 2.5 mm^3^) and Maris Widgeon (35.6 ± 1.5 mm^3^) (*P* > 0.05, Fig. 3d).

### Spikelet morphometric traits of two wheat varieties

**Fig. 4:**
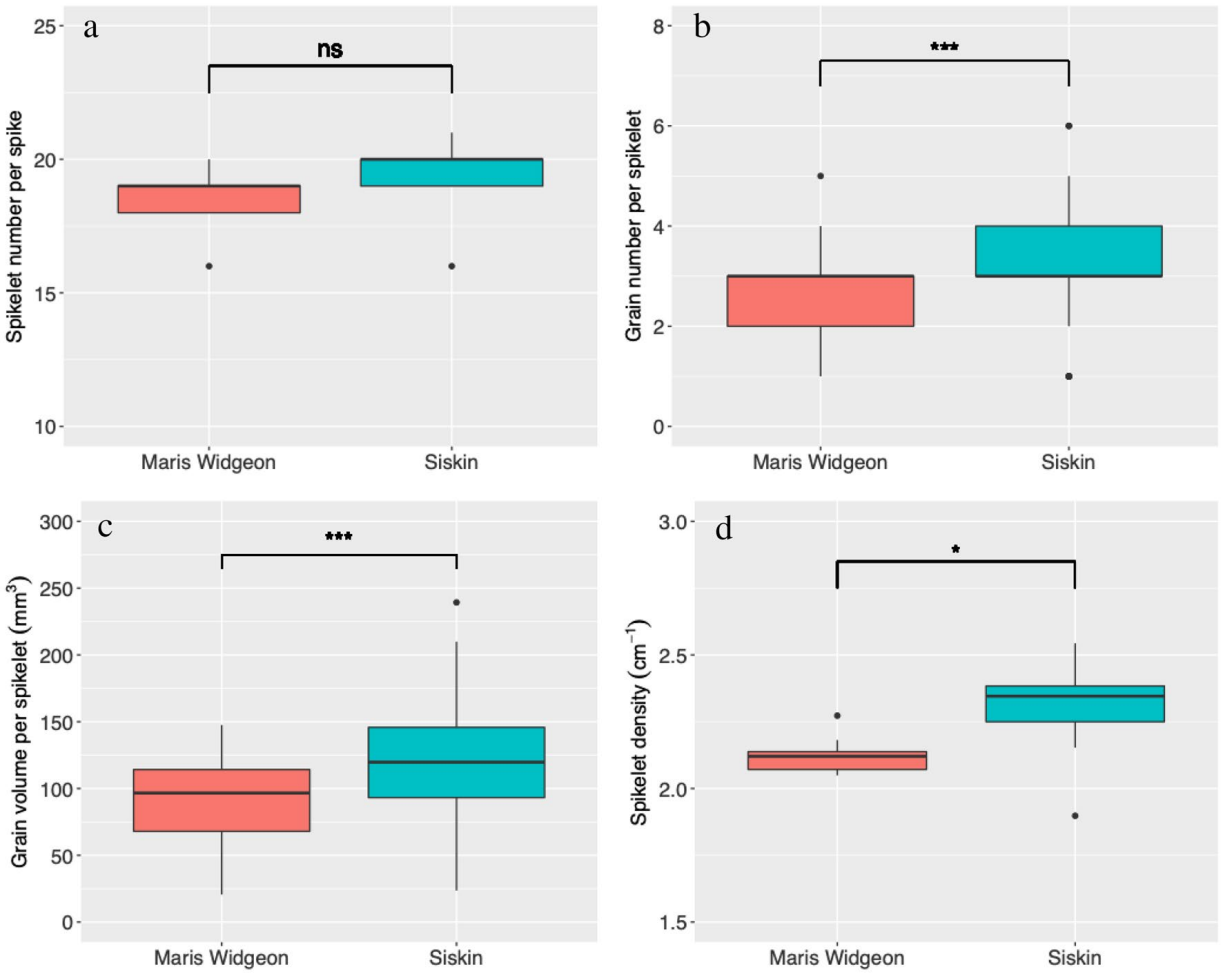
Morphometric traits of the spikelet of the two wheat varieties. **a** spikelet number per spike, **b** number of grains per spikelet, **c** grain volume per spikelet, **d** spikelet density. *ns* not significant, *P* > 0.05; *, *P* < 0.05; ***, *P* < 0.001

The spikelet number per spike did not differ between Maris Widgeon (19.4 ± 1.4) and Siskin (18.4 ± 1.1) (*P* > 0.05, Fig. 4a). However, the average grain number per spikelet of Siskin (3.3 ± 1.0) was significantly greater than that of Widgeon (2.5 ± 0.8) (*P* < 0.001, Fig. 4b). As there were more grains per spikelet, the volume of grains per spikelet of Siskin was 32.2% higher than Maris Widgeon (*P* < 0.001, Fig. 4c). Although spike length and number of spikelets were statistically not different between the two varieties, the spikelet density of Siskin (2.3 ± 0.2 cm^−1^) was significantly higher than that of Maris Widgeon (2.1 ± 0.1 cm^−1^) (*P* < 0.05, Fig. 4d).

**Fig. 5:**
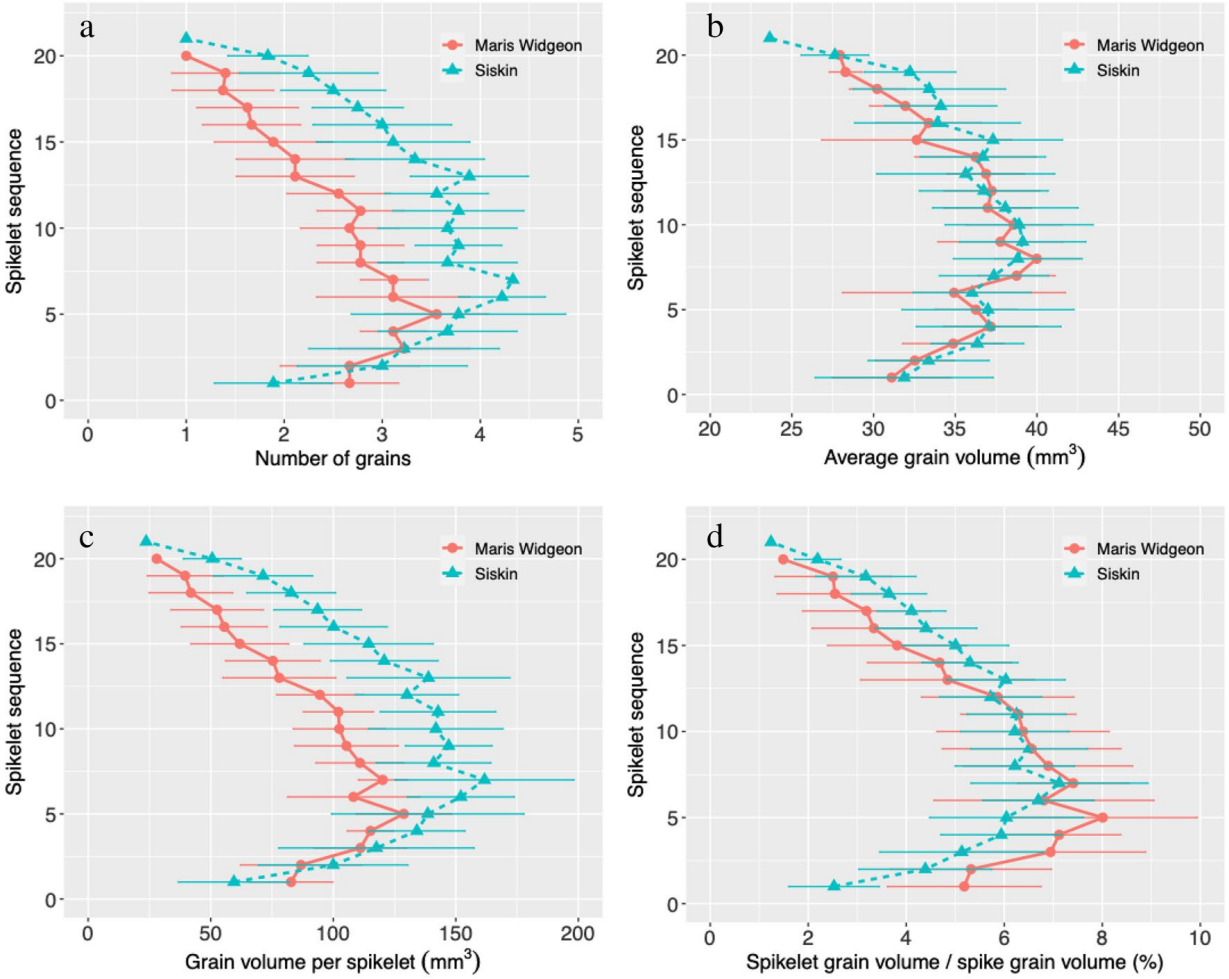
Morphometric traits of the spikelets of the two wheat varieties. **a** number of grains per spikelet, **b** average grain volume per spikelet, **c** grain volume per spikelet, and **d** spikelet grain volume/spike grain volume. Error bars indicate standard deviation

Detailed information on the distribution of spikelet traits along the spike was acquired with CT imaging. The number of grains per spikelet was different at different positions of the spike, with high values observed near the middle part of the spike (Fig. 5a). The same trend was observed for the average grain volume and total grain volume per spikelet (Fig. 5b, c). Grain number per spikelet between Maris Widgeon and Siskin was not different from spikelet 1 to 5, however Siskin had more grains per spikelet from spikelet 6 to 19 (Fig. 5a). The average grain volumes of the spikelets along the spike showed no significant difference between the two varieties (Fig. 5b). Grain volume per spikelet showed a similar trend as the number of grains per spikelet (Fig. 5c). The distribution of the contribution of the spikelet to the spike yield, i.e. grain volume of the spikelet to the total grain volume of the spike, is shown in Fig. 5d. Spikelets 1–5 of Maris Widgeon contributed more than the equivalent spikelets of Siskin, while spikelet 13 and above showed an opposite trend, with spikelets in between showing no difference.

## Discussion

### Assessment of the X-ray CT imaging approach to determine wheat spikelet and spike traits

Wheat spikelets are an important yield component but quantification of their morphometric traits has previously relied on manual measurement [6, 7, 18], which is destructive and very time-consuming. Using X-ray CT imaging, we developed a method that can non-destructively and accurately identify wheat spikelets and quantify their morphometric traits (Figs. 1, 2, 3, 4). Together with the determination of wheat spike and grain traits (Fig. 3) [3–5, 13, 16], a comprehensive range of morphometric traits of wheat spikes and spikelets can be rapidly obtained using X-ray CT imaging. This provides the opportunity to link detailed wheat spike and spikelet traits to agronomic practices and genetic studies supporting future breeding programmes [12, 18].

One previous weakness of X-ray CT imaging for studies of this kind has been the long scanning time, which makes measurement of large number of samples, often important for work of this kind, impossible. For example, it took around 30–40 min to scan one wheat spike in several previous studies [3–5, 13], with the spatial resolution between 15 and 68.8 μm. In this study, we improved on the scanning efficiency through two approaches. The first was by scanning multiple wheat spikes simultaneously using a sample holder containing 9 spikes that were clearly separated, thus avoiding difficulties e.g. separating spikes for subsequent image analysis. A compromise of scanning a large sized sample was a reduction of spatial resolution, i.e. 77 μm in this study. The second approach was using the fast scanning mode of the CT scanner with one scan (9 samples) taking only 5 min. With the chosen scanning settings (e.g. spatial resolution and fast scan), the quality of the reconstructed images was sufficient for the separation of wheat grains and spikelets and the subsequent quantification of their morphometric traits (Fig. 1). The Image processing steps require user interaction when separating the 9 wheat spikes of each scan, while other image analysis procedures can be done automatically, with an average processing time of about 5 min for each spike on a personal PC (CPU3.30 GHz, RAM128GB). This efficient scanning and image analysis methods demonstrates the potential of X-ray CT imaging to determine wheat spikelet and spike traits, even for large-scale experiments.

### X-ray CT imaging revealed different spikelet traits of two wheat cultivars

We compared spikelet traits of two different wheat varieties, a tall, older type, Maris Widgeon and a modern dwarf plant, Siskin. Siskin had a better yield performance than Maris Widgeon, which can be partially attributed to the greater grain volume/mass per spike of Siskin (Fig. 3c). The grain volume per spike is a function of average grain volume and number. As the average grain volume was not different between the two varieties (Fig. 3b), we can conclude Siskin had a higher yield partially because of higher number of grains per spike than Maris Widgeon (Fig. 3d). Previous studies have also revealed an increase of grain numbers is a main cause of grain yield improvement in the last century [2, 10].

Detailed analysis of the spike morphometric traits uncovered new information about the yield components. We found the number of spikelets per spike was not different between the two varieties (Fig. 4a). Therefore, and importantly, the increased number of grains per spike of Siskin was due to the increased number of grains per spikelet (Fig. 4b). The difference in number and volume of grains per spikelet varied with spatial position, with Siskin showing higher values on the upper part of the spike (Fig. 7a, c). A similar trend was also observed for the contribution of spikelet yield to the spike yield (Fig. 5d). This suggest that efforts to increase the number of gains per spikelet at specific positions might be a fruitful avenue to increase wheat grain yield. However, because the two wheat varieties were grown under one specific field condition, we must consider that their performance might be different at other locations, in different growing years or under different agronomic management practices. Future studies that utilize this new method are needed to better understand the relationship between wheat spikelet traits and yields.

## Conclusions

We developed a new method to non-destructively quantify the morphometric traits of wheat spikes and spikelets based on images from X-ray CT scanning. The method can efficiently and accurately determine the number of spikelets per spike, number and volume of grains per spikelet, number and volume of grains per spike, and importantly the spatial distribution of spikelet along the spike, etc. By comparing one modern and one older wheat variety, we found that their spikelets showed different morphometric traits, which can provide useful information for agronomic management and genetic studies to improve wheat grain yield underpinning future breeding programmes.

## Methods

### Field experiment and sampling of wheat spikes

The field experiment was conducted at Rothamsted Experimental Farm, Harpenden, UK. Twenty UK wheat lines, mainly modern elites, were grown using standard farm practices. All treatments had threefold replication, with each treatment grown in each of three randomised blocks following a split plot design. Each plot was 9 by 1.8 m, with a 2 m area at the end of each plot reserved for in-season sampling. The wheats were sown on 9th October 2018 and harvested on 1st and 2nd September, 2019. The spikes were collected at the ripening stage on 8th August 2019. For this study two contrasting lines were chosen, i.e. Maris Widgeon and Siskin. Maris Widgeon, dating from 1964, is now considered outdated whereas Siskin, introduced in 2016, is a current UK commercial variety. Ten spikes were collected from the sampling area of each replicate plot, and were cut just below the spike.

### X-ray CT scanning and image reconstruction

**Fig. 6:**
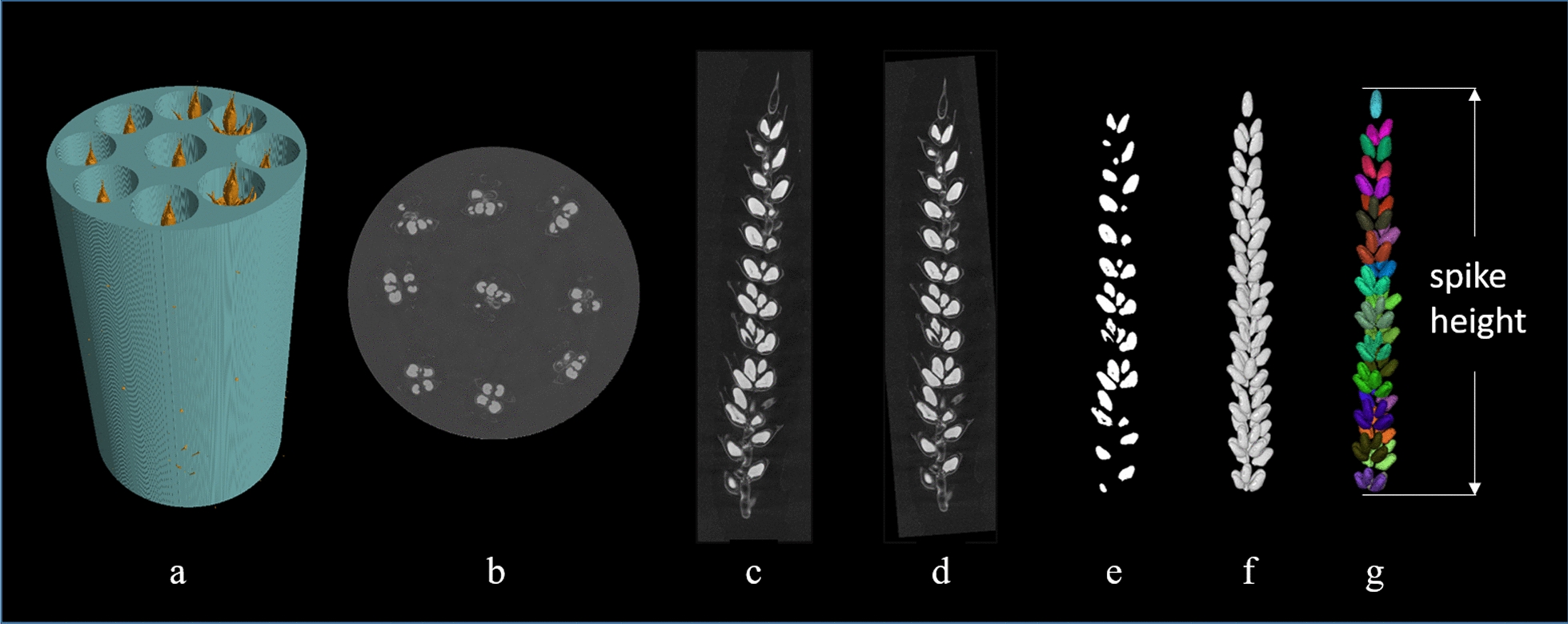
Example of the CT scanning and image processing pipeline for wheat spikes. **a** sample holder with 9 wheat spikes, **b** cross-section of the reconstructed CT volume, **c** longitudinal section of a separated wheat spike, **d** re-orientated wheat spike, **e** segmentation of the grains, **f** 3D visualization of grains, **g** visualization of wheat spikelet with grains in the same spikelet showing the same colour

Wheat spikes were scanned using a *v*|tome|x M 240 kV X-ray CT scanner (GE Sensing and Inspection Technologies, Wunstorf, Germany) at the Hounsfield Facility, University of Nottingham. Three spikes were randomly selected from the collected samples of each plot and a total of 18 spikes were scanned. Nine wheat spikes were separately placed inside cylindrical holes made inside a foam material holder (diameter 10 cm, height 10 cm) in a vertically orientation (Fig. 6a). The holder was fixed on the specimen stage of the scanner and the nine wheat spikes were scanned simultaneously. The voltage and current were set at 85 kV and 120 µA, respectively. A spatial resolution of 77 μm was used in all scans. During the scan, the specimen stage rotated through 360 degrees at a rotation step increment of 0.6° collecting a total of 600 projection images. Exposure time of each projection image was 500 ms and each scan took about 5 min. Reconstruction was conducted using the *phoenix datos|x* software (GE Sensing and Inspection Technologies, Wunstorf, Germany), resulting in a 3D 16-bit greyscale volume. Each XY slice in the volume had a size of 1400 × 2024 voxels, and the length (Z) of the volume was 1600 voxels.

### Image analysis and feature extraction

The 3D volume files were imported into the VG Studio 3.1 software (Volume Graphics GmbH, Heidelberg, Germany). Each of the nine wheat spikes (Fig. 6b) in the same image stack were separated by cropping the corresponding cylindrical columns (Fig. 6c). The inclined wheat spikes (Fig. 6c) were adjusted to a vertical orientation (Fig. 6d) before further processing. The processed 3D volume files of the spikes were exported as 16-bit grayscale slices (tiff format).

Wheat grains were segmentation from the background (Fig. 6e) using the Otsu method [8]. A 3D median filter (size = 3) was used to remove the noises after segmentation. The segmentation and filtering were conducted using the ImageJ software. Some wheat grains were connected and they were separated by the 3D watershed method under the Matlab platform (R2018a). Detailed description and the source codes of the 3D watershed method can be found in Hughes et al. [3]. The grains of the wheat spike were visualized in 3D (Fig. 6f) using the 3D Viewer plugin in ImageJ software.

**Fig. 7:**
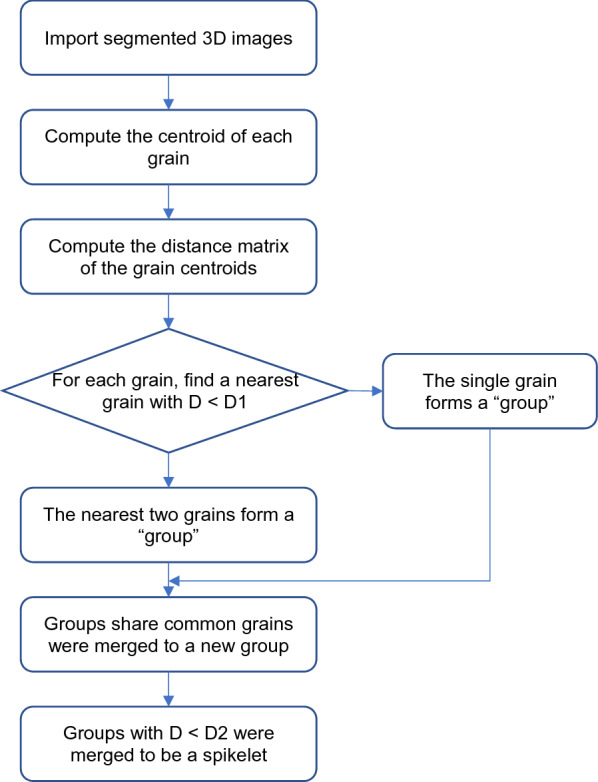
Workflow diagram to determine wheat spikelets of the spikes using the binarized CT images. D1 and D2 are user defined parameters

A program was developed using Matlab to quantify spikelet traits. Wheat spikelets were identified by clustering nearby grains based on their Euclidean distance and relative position, and detailed procedures are presented in Fig. 7. Wheat spikelets were visualized using the 3D Viewer and the 3D Roi Manager plugin [9], with grains belong to the same spikelet shown in the same colour (Fig. 6g). The Matlab code for the spikelet identification and ImageJ macro for spiklet visualization are provided as supplemental material (Additional file 1: S1, 2).

The number, volume and coordinates of the centroid of the grains were determined using the “regionprops” function in the Image Processing Toolbox of MATLAB. The spike height was determined as the distance between the top and the bottom grains as shown in Fig. 6g. The number of spikelets per spike and number and volume of grains per spikelet were determined using a Matlab script. The vertical distribution of spikelets was also determined, with the spikelet sequence labelled from bottom to top of the spike (Fig. 2). Spikelet density was determined as the number of spikelets per cm of spike length [6].

### Validation of the CT imaging-based method

The number of grains per spike, number of spikelets per spike and number of grains per spikelet were manually counted and compared with the data derived from CT image analysis. The weight of grains of each spike was measured and their correlation with the image-based volume of the grains were determined.

### Statistical analysis

Statistical analysis was performed using RStudio software (R version 3.3.3). The normality of residuals and the homogeneity of variances of the data was checked by the Shapiro–Wilk test and the Levene's test respectively. One-way ANOVA was conducted to compare the means of spikelet traits between the two wheat varieties. Correlation analysis was performed using Pearson’s correlation coefficients. Data in the results section were presented as mean ± standard deviation.

## Supplementary Information

**Additional file 1**: **S1**. Matlab code for the spikelet identification.

**Additional file 2**: **S2**. ImageJ macro for the spikelet visualization.

## Data Availability

All data generated or analysed during this study are included in this published article [and its supplementary information files].

## Abbreviations

CT: Computed tomography
3D: Three-dimensional
